# Take -home naloxone rescue kits following heroin overdose in the emergency department to prevent opioid overdose related repeat emergency department visits, hospitalization and death- a pilot study

**DOI:** 10.1186/s12913-019-4734-5

**Published:** 2019-12-11

**Authors:** Joan Papp, Mayur Vallabhaneni, Ariel Morales, Jon W. Schrock

**Affiliations:** Cleveland, USA

**Keywords:** Heroin, Opioid, Overdose, Project DAWN, Naloxone, Mortality, Emergency department

## Abstract

**Background:**

Opioid overdoses are at an epidemic in the United States causing the deaths of thousands each year. Project DAWN (Deaths Avoided with Naloxone) is an opioid overdose education and naloxone distribution program in Ohio that distributes naloxone rescue kits at clinics and in the emergency departments of a single hospital system.

**Methods:**

We performed a retrospective analytic cohort study comparing heroin overdose survivors who presented to the emergency department and were subsequently discharged. We compared those who received a naloxone rescue kit at discharge with those who did not. Our composite outcome was repeat opioid overdose related emergency department visit(s), hospitalization and death at 0–3 months and at 3–6 months following emergency department overdose. Heroin overdose encounters were identified by ICD- 9 or 10 codes and data was abstracted from the electronic medical record for emergency department patients who presented for heroin overdose and were discharged over a 31- month period between 2013 and 2016. Patients were excluded for previous naloxone access, incarceration, suicidal ideation, admission to the hospital or death from acute overdose on initial emergency department presentation. Data was analyzed with the Chi- square statistical test.

**Results:**

We identified 291emergency department heroin overdose encounters by ICD-9 or 10 codes and were analyzed. A total of 71% of heroin overdose survivors received a naloxone rescue kit at emergency department discharge. Between the patients who did not receive a naloxone rescue kit at discharge, no overdose deaths occurred and 10.8% reached the composite outcome. Of the patients who received a naloxone rescue kit, 14.4% reached the composite endpoint and 7 opioid overdose deaths occurred in this cohort. No difference in mortality at 3 or 6 months was detected, *p* = 0.15 and 0.36 respectively. No difference in the composite outcome was detected at 3 or 6 months either, *p* = 0.9 and 0.99 respectively.

**Conclusions:**

Of our emergency department patients receiving a naloxone rescue kit we did not find a benefit in the reduction of repeat emergency department visits hospitalizations, or deaths following a non-fatal heroin overdose.

## Background

The United States is facing an epidemic of opioid overdose deaths of unprecedented scale [1–3]. Since 2000, our nation has seen a 137% increase in the rate of overdose related death, including a 200% increase in opioid related overdose death rate [2]. The momentum of this epidemic has not slowed with 70, 237 overdose deaths in 2017 and of these deaths, two thirds were attributed to an opioid [4, 5]. The New England and Midwestern states are among the most devastated regions of the country, with Ohio reporting a 98% increase in unintentional deaths between 2010 and 2015 [6].

The opioid crisis has spanned nearly three decades and has progressively become more deadly as synthetic opioids have become widely available [4]. The epidemic started in the mid-1990s with the increased availability of prescription opioid pain relievers [1, 3, 7]. Many opioid dependent users transitioned to heroin as a more cost-effective alternative to prescription opioids, which were becoming more difficult to obtain beginning in 2010 [4, 7, 8]. This transition was first described in a study of heroin users entering drug treatment in the last decade in which 75% reported that they were first introduced to heroin from prescription drugs [7]. This substitution of prescription opioids for heroin led to a tripling of overdose deaths over a four-year period [2]. In 2013, illicitly manufactured fentanyl (IMF), a purely synthetic opioid 50–100 times more potent than morphine emerged and continues to be the greatest current threat to opioid users [9]. IMF has been identified in combination with heroin, cocaine or as pure IMF [3, 10–12]. The rate of fatal overdose from fentanyl and its analogs have doubled each year between 2013 and 2016 in all age, gender and ethnic subgroups in the U.S. [12, 13] In Cuyahoga County, the unintentional overdose death rate per 100,000-population increased between 2013 and 2016 from 20.2 to 43.6 respectively, fueled by emergence of IMF. Statewide, overdose fatalities in Ohio attributed to fentanyl increased steadily as well: 4% in 2013, 19.9% in 2014, 37.9% in 2015 and 58.2% in 2016.

Risk factors contributing to fatal opioid overdose have been identified by other investigators and include: male gender, history of injection heroin use, a period of recent abstinence (commonly due to inpatient drug treatment or jail), mixing drugs, history of previous opioid overdose(s) and older age [14, 15].

Patients who use opioids frequently seek care in the emergency department (ED) allowing for a unique opportunity to offer novel interventions to reduce mortality and improve outcomes in this high- risk population [16, 17]. In a study of ED patients who survived opioid overdose, the one-year mortality rate was 5.5% with the highest risk of subsequent fatality occurring in the first month after overdose [18]. Interventions directed at reducing this early mortality that have shown promise include ED-initiated buprenorphine/naloxone treatment, access to a peer coaches, and take-home naloxone [19, 20]. Of these interventions, only ED initiated buprenorphine/naloxone has been rigorously evaluated [21, 22]. It is likely that additional interventions may also contribute to improved outcomes either alone or in combination. Without intervention in the ED, patients are likely to continue drug use and maintain a high risk for future fatal overdose.

Naloxone Hydrochloride is an opioid antagonist, which reverses the respiratory, and CNS depression of opioid overdose and can prevent death. Naloxone has been FDA approved for intravenous, intramuscular (IM) injection or intranasal (IN) administration [23–25]. The opioid antidote is available in the United States with a prescription, is not a controlled substance and has no potential for abuse.

While EDs and EMS providers frequently administer naloxone to reverse opioid overdose, until recently, laypersons did not readily have access to the antidote to revive overdose victims [26]. It has been shown that providing take-home naloxone rescue kits (NRKs) to at-risk patients reduces opioid overdose death rates in communities that implement opioid education and naloxone distribution programs and is cost effective under highly conservative estimates [27, 28]. The optimal amount of training required to competently respond to an overdose with naloxone is unknown, however both trained and untrained rescuers respond similarly when responding to an overdose suggesting that minimal education is required [29]. It has also been shown that providing naloxone rescue kits to patients in the ED is a feasible practice but still this potentially lifesaving intervention is not standard care in most E.Ds [19, 20, 30]. No studies to date have evaluated patient level outcomes in persons who receive NRKs in the ED.

## Methods

### Setting

This retrospective analytic cohort study took place at an urban academic medical center in Northeast Ohio with a main campus ED with 100, 000 annual visits. This same institution operates Cuyahoga County Project DAWN, *Deaths Avoided with Naloxone*, a hospital- based opioid education and naloxone distribution (OEND) program. DAWN was created to educate opioid users and other rescuers on risk factors, recognition and response to opioid overdose. The program distributes NRKs and was the sole point of access for take- home naloxone during this study period. Cuyahoga County Project DAWN is unaffiliated with the Drug Awareness Warning Network (DAWN) the public health surveillance system, managed by SAMHSA. In addition to the EDs of this medical center, project DAWN provides naloxone rescue kits (NRKs) at community walk- in sites, the Cuyahoga County Corrections Center (CCCC), and the inpatient units throughout this institution. Through our electronic medical record (EPIC, Verona WI), we were able to record all patient encounters for distribution of NRKs in the electronic health record of this single institution.

Prior to implementation of the naloxone distribution program in the ED, all providers, nurses and paramedics were educated with a mandatory educational module on the benefits of take home naloxone as well as the procedure for dispensing NRKs in the ED. A hospital wide policy for naloxone distribution was created for ordering take-home naloxone and was available to all providers and nurses. One year after ED program implementation, providers were surveyed on their knowledge and attitudes toward take-home naloxone in the ED and 66.7% of providers responded positively to the statement “*This is a lifesaving intervention that I support*.”

### Participants

Adult patients between 18 and 89 years of age with an ICD-9 or 10 diagnosis of heroin overdose treated in the ED who were subsequently discharged were reviewed. Patients were excluded from further review if there was documentation of the following: previous NRK access, prescription for naloxone, incarceration while in the ED, reported suicidal ideation, admission to the hospital or death from acute overdose on initial ED presentation.

We evaluated the effect of take-home NRKs in adult ED patients presenting for heroin overdose who were subsequently discharged over a 31- month period between September 1, 2013 and April 1, 2016. For each eligible patient encounter following an ED non-fatal presentation, the composite outcome of repeat opioid overdose related ED visit(s), hospitalization, and death were recorded at 0–3 months and 3–6 months. Demographics and medical history for each patient were also recorded. At the first ED visit for heroin overdose, we recorded whether the patient received an NRK or not. Deaths were tracked by a subjects’ presentation to the Cuyahoga County Medical Examiner. Patients who received an NRK at ED discharge were compared to those who did not to determine if a statistically significant difference occurred. This study was reviewed, and a waiver of informed consent was approved by our hospital’s Institutional Review Board.

#### Measurement and data collection

ED heroin overdose encounters were initially identified by obtaining a report of all overdose related ICD-9 or 10 codes during the study period. Two research assistants with a bachelor’s degree education manually reviewed all encounters. Research assistants were trained to abstract data from the medical record prior to the start of the study using a set of “practice” medical records. The research assistants were trained on the abstraction protocol, case selection and inclusion/exclusion criteria. Each variable was explicitly defined as follows: independent variable was defined as receiving an NRK or not receiving an NRK at ED discharge. Dependent variables are opioid overdose related repeat ED visit(s), hospitalization(s) or death. Extraneous variables that were examined include: age, gender, zip code, refusal of naloxone rescue kit, history of past documented opioid overdose, recent period of abstinence, medical conditions, prescription medications, use of other illicit drugs and route of heroin administration as documented in the electronic health record. The data from the electronic chart was collected using a standardized abstraction form and entered into the Redcap secure database. Inter-rater agreement was calculated periodically throughout data collection from a sample of charts. Each research assistant was blinded to the results of the original abstractor. Performance of abstractors was monitored periodically throughout the study and inter-rater agreement was monitored and feedback on performance was given. Routine meetings were held to adjudicate conflicts and any data that was conflicting, missing, ambiguous or unknown. It was not practical to blind the abstractors to the purpose of the study.

### Sample size calculation

We utilized a composite outcome of opioid overdose related repeat ED visits, hospitalization and mortality after ED discharge from opioid overdose. Limited data from the literature were available to accurately calculate a sample size in this population. Assuming a baseline incidence of the composite outcome of 5% and a treatment group incidence of 1%, a sample size of 284 patients in each group would be required to detect an effect size of 0.04 and provide a power of 80% with a type I error rate (alpha) of 5%.

### Data analysis

Data are presented as frequencies and was analyzed with the Chi- square statistical test using Stata (State College, TX) statistical software.

## Results

During the study period we identified 397 heroin overdoses, 69 patients met one or more exclusion criteria, 37 patients had missing or incomplete data and 291 were analyzed. Patients were excluded from further review if one or more of the following exclusion criteria were present: documentation of previous access to or prescription for naloxone (36), incarceration at time of initial ED overdose [4], suicidal ideation [9], admitted to the hospital at time of initial heroin overdose [31] or death [1].

The median age of analyzed patients was 34 years (IQR) and 70% were male. No ethnicity data was available for review. The following medical conditions were reported among all patients included for review: hypertension 17.1%, asthma/COPD 26.4%, diabetes 7.4%, hepatitis C 40.3%, HIV 2.8%, cardiac disease 13.6%, psychiatric disease 60.2%, other medical condition (19.4%). We found 74% reported a marital status of single, 13.5% married and 12.1% divorced. A total of 13.1% of patients had a current opioid prescription for analgesia, 2.9% had a current prescription for a benzodiazepine and 1.2% had a current prescription for an opioid for medication assisted treatment (MAT) of opioid use disorder (OUD). Prior opioid overdoses were recorded in 31.4% of all ED overdose survivors with 31% reporting a period of abstinence greater than 1 week prior to date of initial ED overdose during the study period.

### Main results

Over the study period 71% of heroin overdose survivors received an NRK at ED discharge. A total of 39 unique patients reached one or more of the composite outcome measures recorded. Among the patients who did not receive an NRK at discharge, 38% were offered an NRK but refused it and 15% of these patients stated that they already had access to an NRK. No overdose deaths occurred among patients who did not receive an NRK and 10.8% reached the composite outcome. 14.4% of patients who received an NRK reached the composite endpoint and 7 opioid overdose deaths occurred in this cohort. Cause of death for each decedent is listed by month and year in Table 1. No deaths occurred in 2013 or 2014, and fentanyl or a fentanyl analog contributed to all deaths occurring in 2016. No difference in mortality at 3 or 6 months was detected, *p* = 0.15 and 0.36 respectively. No difference in reaching the composite outcome was detected at 3 or 6 months either, *p* = 0.9 and 0.99 respectively. Composite outcomes and mortality at 0–3 and 3–6 months are listed in Table 2.

**Table 1:** Cause of Death by Month and Year

Month of Death	Year of Death	Cause of Death Listed in Medical Examiner’s report
January	2015	Heroin, cocaine
April	2015	Heroin, fentanyl
June	2015	Oxycodone
March	2016	Fentanyl, heroin
June	2016	Heroin, fentanyl, acetyl fentanyl, cocaine and diphenhydramine
August	2016	Heroin, furanyl fentanyl, alprazolam, and diphenhydramine
September	2016	Heroin, fentanyl

**Table 2:** Composite outcomes at 0–3 and 3–6 months, n (%)

NRK	Death at 0–3 months	Death at 3–6 months	Total Deaths	Composite Outcome 0–3 months	Composite Outcome at 3–6 months	Total Composite Outcome
No NRK 28.5% (*n* = 83)	0	0	0	5 (6)	4 (4.8)	9 (10.8)
Received NRK 71% (*n* = 208)	5 (2.4)	2 (0.9)	7 (3.3)	16 (7.6)	14 (6.7)	30 (14.4)
Total (*n* = 291)	5 (1.7)	2 (0.7)	7 (2.4)	21 (7.2)	20 (6.1)	39 (13.4)

## Discussion

EDs provide critical access for at–risk patients who use opioids and are an ideal setting to introduce novel interventions to reduce morbidity and mortality in this population. Although numerous interventions are currently being utilized, few have been rigorously studied to determine effectiveness. In this study, we were unable to detect a significant difference in patient level outcomes for overdose survivors who received an NRK at ED discharge. In a previous study by Walley, et al. opioid death rates were reduced in the communities that implemented OEND programs. This is likely explained by the observation that patients in their study reported that they used their naloxone for someone else (friend, stranger or partner/family) in 93% of the rescues, i.e. most people used their NRK to save someone other than themselves [28]. Hence, although a reduction in mortality rate in the community was reported, individual patient -level outcomes were not measured or reported.

In our study, it is notable that all fatalities and most patients who reached the composite outcome were in the group that received an NRK at ED discharge. This finding may be due to the greater proportion of overdose survivors (71%) that received an NRK. NRK distribution became standard care for overdose aftercare in our ED during the study period and increased over time (Table 3). In 2016, when ED overdose mortality peaked, 83% of overdose survivors were discharged with an NRK. Another factor that likely influenced this result is the overall increase in overdose mortality in Cuyahoga County during the study period (Table 4), which was driven by the introduction of more lethal IMF into the illicit drug market. This countywide change in cause of death was reflected in the overdose cause of death noted in our ED fatalities as well (Table 1).

**Table 3:** Year of ED NRK distribution between September 1, 2013 and April 1, 2016

Year	Total NRK received (%)	Total NRK not received (%)
2013	0	14 (100)
2014	53 (71)	21 (28)
2015	75 (70)	32 (30)
2016	80 (83)	16 (17)

**Table 4:** Cuyahoga County Overdoses 2013–2016

Year	Drug	Total Deaths
2013	Heroin	194
	Fentanyl	5
	Other opioids	101
2014	Heroin	198
	Fentanyl	37
	Other opioids	87
2015	Heroin	184
	Fentanyl	92
	Other opioids	80
2016	Heroin	320
	Fentanyl	399
	Other opioids	99

Adapted from Medical Examiner’s Final Drug Deaths Report 2016 [31]

While our county was experiencing this dramatic spike in overdose fatalities (Table 4), we report a more modest increase in overall mortality year to year in our ED population (Table 1). Future investigations should be designed to further evaluate this trend and to determine which if any protective factors exist for patients evaluated in the ED for opioid overdose. Pre-existing protective factors in this population may include a safer drug use environment, availability of family/friends willing to activate EMS, bystander access to NRKs for overdose reversal and closer proximity to hospital. Additional protective factors provided by the ED may include stabilization of other underlying medical conditions, access to additional ED support services and referrals to addiction treatment and primary care.

The need for EDs to play a more active role in caring for patients who use illicit opioids has increased steadily over the past decade and is likely to continue for the foreseeable future. Future investigations are needed to determine which interventions are most effective.

This study adds to our overall knowledge of the characteristics of patients presenting to the ED for presumed heroin overdose in a community impacted predominately by heroin prior to 2015 and fentanyl thereafter. It is the first study to examine patient level outcomes in ED overdose victims who are discharged with an NRK and although the study was underpowered to detect an effect and the reported outcomes did not reach statistical significance, important data was gained that will be integral to the design and application of future investigations.

### Limitations

The total number of patient encounters eligible for inclusion did not reach the calculated sample size; therefore, this retrospective study was underpowered to detect the desired effect size of 0.04. Additionally, our reported outcomes did not reach statistical significance.

Our institution operates all Project DAWN naloxone distribution sites in Cuyahoga County making it possible to determine if a patient has access to a naloxone kit through access to our electronic health record. Every attempt to exclude patients who had prior access to a naloxone kit was made, however it may have been possible that patients obtained a naloxone kit from an entity outside of Cuyahoga County confounding results. To limit the impact of this we reviewed all charts individually to determine if any documentation of prior kit access was noted in ED documentation as well as a review of all prescription medications.

## Conclusions

Our study did not demonstrate a significant benefit in the patient level composite outcome of repeat ED visit, hospitalization and death between patients who received an NRK in the ED following a non-fatal heroin overdose and those who did not. Future investigations are needed to identify effective ED interventions to improve patient level outcomes in overdose survivors.

## Data Availability

The datasets used and/or analyzed during the current study are available from the corresponding author on reasonable request.

## Abbreviations

CCCC: Cuyahoga County Corrections Center
CDC: Centers for Disease Control
CNS: Central nervous system
DAWN: Deaths avoided with naloxone
ED: Emergency department
EMS: Emergency medical systems
FDA: Federal Drug Administration
IM: Intramuscular
IMF: Illicitly manufactured fentanyl
IN: Intranasal
NRK: Naloxone rescue kit
OEND: Opioid education and naloxone distribution

## References

[1] Centers for Disease C, Prevention (2012). CDC grand rounds: prescription drug overdoses - a U.S. epidemic. MMWR Morb Mortal Wkly Rep.

[2] Rudd RA, Aleshire N, Zibbell JE, Gladden RM (2016). Increases in drug and opioid overdose deaths - United States, 2000-2014. MMWR Morb Mortal Wkly Rep.

[3] Seth P, Scholl L, Rudd RA, Bacon S (2018). Overdose deaths involving opioids, cocaine, and psychostimulants - United States, 2015-2016. MMWR Morb Mortal Wkly Rep.

[4] Seth P, Scholl L, Rudd RA, Bacon S (2018). Overdose deaths involving opioids, cocaine, and psychostimulants - United States, 2015-2016. Am J Transplant.

[5] Scholl L, Seth P, Kariisa M, Wilson N, Baldwin G (2018). Drug and opioid-involved overdose deaths - United States, 2013-2017. MMWR Morb Mortal Wkly Rep.

[6] Daniulaityte R, Juhascik MP, Strayer KE, Sizemore IE, Harshbarger KE, Antonides HM (2017). Overdose deaths related to fentanyl and its analogs - Ohio, January-February 2017. MMWR Morb Mortal Wkly Rep.

[7] Cicero TJ, Ellis MS, Surratt HL, Kurtz SP (2014). The changing face of heroin use in the United States: a retrospective analysis of the past 50 years. JAMA Psychiat.

[8] Jones CMLJ, Gladden RM, Bohm MK (2015). Vital signs: demographic and substance use trends among heroin users - United States, 2002-2013. MMWR Morb Mortal Wkly Rep.

[9] Spencer MR WM, Bastian BA, Trinidad JP, Hedegaard H. Drug overdose deaths involving fentanyl, 2011–2016. National Vital Statistics Reports; vol 68 no 3. Hyattsville: National Center for Health Statistics. 2019. 31112123

[10] Increases in Fentanyl Drug Confiscations and Fentanyl-Related Overdose Fatalities. Atlanta, Georgia2015 [updated October 26, 2015. Official CDC Health Advisory]. Available from: www.emergency.cdc.gov/han/han00384.asp.

[11] Gladden RM, Martinez P, Seth P (2016). Fentanyl law enforcement submissions and increases in synthetic opioid-involved overdose deaths - 27 states, 2013-2014. MMWR Morb Mortal Wkly Rep.

[12] Spencer MR, Warner M, Bastian BA, Trinidad JP, Hedegaard H (2019). Drug overdose deaths involving fentanyl, 2011-2016. Natl Vital Stat Rep.

[13] Hedegaard H, Bastian BA, Trinidad JP, Spencer M, Warner M (2018). Drugs Most frequently involved in drug overdose deaths: United States, 2011-2016. Natl Vital Stat Rep.

[14] Hickman M, Carnwath Z, Madden P, Farrell M, Rooney C, Ashcroft R (2003). Drug-related mortality and fatal overdose risk: pilot cohort study of heroin users recruited from specialist drug treatment sites in London. J Urban Health.

[15] Strang J (2015). Death matters: understanding heroin/opiate overdose risk and testing potential to prevent deaths. Addiction..

[16] Bahorik AL, Satre DD, Kline-Simon AH, Weisner CM, Young-Wolff KC, Campbell CI (2018). Alcohol, marijuana, and opioid use disorders: 5-year patterns and characteristics of emergency department encounters. Subst Abus.

[17] John WS, Wu LT (2019). Sex differences in the prevalence and correlates of emergency department utilization among adults with prescription opioid use disorder. Subst Use Misuse.

[18] Weiner SG, Baker O, Bernson D, Schuur JD (2019). Ann Emerg Med: One-Year Mortality of Patients After Emergency Department Treatment for Nonfatal Opioid Overdose.

[19] Samuels EA, Baird J, Yang ES, Mello MJ. Adoption and Utilization of an Emergency Department Naloxone Distribution and Peer Recovery Coach Consultation Program. Acad Emerg Med. 2019;26(2):160–73. 10.1111/acem.13545 30074673

[20] Samuels E (2014). Emergency department naloxone distribution: a Rhode Island department of health, recovery community, and emergency department partnership to reduce opioid overdose deaths. R I Med J (2013).

[21] D'Onofrio G, Chawarski MC, O'Connor PG, Pantalon MV, Busch SH, Owens PH (2017). Emergency department-initiated buprenorphine for opioid dependence with continuation in primary care: outcomes during and after intervention. J Gen Intern Med.

[22] D'Onofrio G, O'Connor PG, Pantalon MV, Chawarski MC, Busch SH, Owens PH (2015). Emergency department-initiated buprenorphine/naloxone treatment for opioid dependence: a randomized clinical trial. JAMA..

[23] Doe-Simkins M, Walley AY, Epstein A, Moyer P (2009). Saved by the nose: bystander-administered intranasal naloxone hydrochloride for opioid overdose. Am J Public Health.

[24] Loimer N, Hofmann P, Chaudhry HR (1994). Nasal administration of naloxone is as effective as the intravenous route in opiate addicts. Int J Addict.

[25] Wermeling DP (2013). A response to the opioid overdose epidemic: naloxone nasal spray. Drug Deliv Transl Res.

[26] Wheeler E, Jones TS, Gilbert MK, Davidson PJ (2015). Opioid overdose prevention programs providing naloxone to laypersons - United States, 2014. MMWR Morb Mortal Wkly Rep.

[27] Coffin PO, Sullivan SD (2013). Cost-effectiveness of distributing naloxone to heroin users for lay overdose reversal. Ann Intern Med.

[28] Walley AY, Xuan Z, Hackman HH, Quinn E, Doe-Simkins M, Sorensen-Alawad A (2013). Opioid overdose rates and implementation of overdose education and nasal naloxone distribution in Massachusetts: interrupted time series analysis. BMJ..

[29] Doe-Simkins M, Quinn E, Xuan Z, Sorensen-Alawad A, Hackman H, Ozonoff A (2014). Overdose rescues by trained and untrained participants and change in opioid use among substance-using participants in overdose education and naloxone distribution programs: a retrospective cohort study. BMC Public Health.

[30] Dwyer K, Walley AY, Langlois BK, Mitchell PM, Nelson KP, Cromwell J (2015). Opioid education and nasal naloxone rescue kits in the emergency department. West J Emerg Med.

[31] Gilson T (2016). Final drug deaths report.

